# Receptivity and Preferences for Lifestyle Programs to Reduce Cancer Risk among Lung Cancer Family Members

**DOI:** 10.4172/2472-0429.1000110

**Published:** 2016-06-20

**Authors:** Lisa A Howell, Tabetha A Brockman, Pamela S Sinicrope, Christi A Patten, Paul A Decker, Allan Busta, Shawn Stoddard, Sheila R McNallan, Ping Yang

**Affiliations:** 1 Department of Psychology and Psychiatry, Mayo Clinic, Rochester, MN 55905, USA; 2 Behavioral Health Research Program, Mayo Clinic, Rochester, MN 55905, USA; 3 Department of Medical Genetics, Mayo Clinic, Rochester, MN 55905, USA; 4 Department of Health Sciences Research, Mayo Clinic, Rochester, MN 55905, USA

**Keywords:** Lung cancer, Family, Health behavior change, Perceived risk, Risk reduction, Lifestyle

## Abstract

**Background:**

Lifestyle factors and genetic information has been found to contribute to the occurrence of lung cancer. This study assessed receptivity to participating in lifestyle programs to reduce cancer risk among unaffected lung cancer family members. We also explored demographic, medical, and psychosocial correlates of willingness to participate in lifestyle programs.

**Methods:**

Family members who are part of a lung Cancer Family Registry were asked to fill out a survey assessing their receptivity to cancer risk reduction programs including preferences for an individual or family-based program.

**Results:**

Of the 583 respondents, 85% were “Somewhat” or “Definitely” willing to participate in a lifestyle program. Among those receptive, about half (56%) preferred a family-based approach. Preferred programs included weight management (36%) and nutritional information (30%). Preferred delivery channels were Internet (45%) and mail-based (29%) programs. On multivariate analysis, those definitely/somewhat receptive reported greater exercise self-efficacy scores (p=0.025).

**Conclusion:**

The majority of the sample was receptive to lifestyle programs that might decrease cancer risk. There was a large preference for family-based weight management and nutritional programs. Further research is indicated to determine how to best incorporate a family-based approach to lifestyle programs for cancer family members.

## Introduction

Lung cancer is the leading cause of cancer-related mortality among both men and women in the United States [1]. Despite advances in early detection, the five-year survival rate for all stages combined remains low (17%) [1–3]. Mortality rates of lung cancer are highest in Non-Hispanic Black males (95.4 per 100,000), followed by Non-Hispanic White males (81.3 per 100,000), followed by American Indian and Alaska Native males (68.5 per 100,000), and finally by Non-Hispanic White females (59.3 per 100,000) [1]. Regional differences exist as well, suggesting that lung cancer mortality is higher in the Midwestern and Southern states, especially for women [4]. This form of cancer has been linked to a high level of morbidity, fatigue, pain, and respiratory difficulties [5].

There has not been one causal risk factor identified to explain the origin of lung cancer, suggesting this disease is the result of complex gene-environment interactions [6]. Given this information, research has been focusing on the identification of risk factors and behavioral changes that can influence the development of this disease. The most preventable risk factor for lung cancer is cigarette smoking [1,7]. Given that cigarette smoking causes approximately 80% to 90% of lung cancer incidences [1], smoking cessation is essential for prevention of lung cancer. Recent studies are linking possible benefits from regular exercise and eating a healthy diet as a role in decreasing one’s risk of developing lung cancer [1,7–14]. However, 15% to 25% of lung cancer-related deaths involve people who have never smoked [6,15,16]. This suggests that genetic, environmental, hormonal, and viral elements are also important factors in the development of this illness [6].

Some individuals may have a greater genetic susceptibility for developing lung cancer [17]. Hindorff et al. [17] found that certain genetic factors can explain approximately 7% of the variance in familiar risk of lung cancer. It is estimated that an individual’s risk of developing lung cancer doubles if he or she has a first degree relative with lung cancer [18–20]. This risk is higher for women than men and increases substantially with more than one affected relative [15,19]. Maternal family cancer history appears to be highly correlated to the development of lung cancer, suggesting that women are more sensitive to possible carcinogens [15]. Research suggests those who are diagnosed at a younger age typically have a genetic marker for this cancer type [1]. To decrease the incidence of unnecessary lung cancer diagnoses, more research is needed to understand family members’ receptiveness, as well as factors associated with engaging in preventative health behaviors.

Behavioral risk factors may play an important role in the development of this cancer for those with a genetic susceptibility. It has been found that those who are identified as at a higher risk for lung cancer via genetic testing would have increased interest in smoking cessation programs; however, those with a lower risk result had neutral attitudes towards smoking cessation [21]. Heightened awareness of an increased risk of cancer due to family history may cause individuals to perceive themselves as being more susceptible to the disease. This heightened awareness, coupled with the fact that cancer risk reducing and promoting behaviors run in families [22], suggests that families with a diagnosis of cancer in a member present logical target for cancer risk reduction interventions. In prior research [23,24], unaffected family members of those with a family history of colon and pancreatic cancer expressed interest in making lifestyle changes in nutrition and weight management to reduce their risk of cancer. Many of these family members preferred to participate in programs with their family or friends, rather than alone. Thus, a diagnosis of lung cancer in the family may serve as a teachable moment [25–27] for families, motivating them to engage in cancer-related health behavior change, either individually or collectively.

Current research is beginning to explore the receptiveness of lung cancer patients and/or family members of lung cancer patients to behavioral health promotion programs to prevent or reduce cancer risk. Bastian et al. [28] found that family members of lung cancer patients who were women, in close geographical location to the patient, and whose patients had late stage disease, were more likely to enroll in smoking cessation programs to reduce their own cancer risk [28]. The enrollment rate into the smoking cessation program within this study, however, remained relatively low; only 38% of those contacted agreed to participate [28]. Witnessing a family member undergo lung cancer treatment may be a motivating factor in smoking cessation for those at increased risk of developing lung cancer. In Butler’s study [29], 72% were interested in smoking cessation programs after having a family diagnosed with lung cancer. Kristeller et al. [30] explored the receptiveness of relatives of cancer patients toward risk and behavioral programs. This study found that family members were receptive to discussing cancer and possible programs, but that spontaneous behavior change was low among family members [30]. Additionally, Schnoll et al. [31] found that family members of lung cancer patients were more likely than other cancers to sign up for a smoking cessation program; however, they had higher rates of discontinuation. These findings highlight that, while family members may be receptive to behavior change efforts, there still is a gap in actual behavioral change. Thus, more information is needed on intervention preferences (exercise, weight management), program format (family versus individual), and delivery channels (web-based versus face-to-face). Also, investigations into aspects of the family cancer experience (e.g., timing of diagnosis to program recruitment) are needed.

The purpose of this study was to explore the feasibility of developing lifestyle interventions targeting lung cancer families. As a logical first step, we assessed members’ likelihood of participating in such a program, as well as preferences for program content and delivery methods. Secondly, we report the association of such receptivity to their current health behaviors as well as demographic, medical, and psychosocial characteristics.

## Materials and Methods

### Participants and procedure

Our study was approved by the Mayo Clinic Institutional Review Board. Participants included at-risk unaffected family members related to the proband through the Mayo Clinic Genetic Epidemiology of Lung Cancer Consortium (GELCC). The GELCC was established in 1997 with funding from the National Institutes of Health and, with ongoing data collection, has accrued over 700 families with three or more first-degree relatives with lung cancer (see http://epi.grants.cancer.gov/Consortia/single/gelcc.html) [32,33]. Eligibility for at-risk unaffected family member participants included family history of two of more relatives affected with lung cancer, over the age 18, able to provide written informed consent, and not incarcerated.

In March 2009, 715 GELCC respondents were mailed a packet including: 1) a cover letter explaining the research study; 2) a consent form; 3) a survey with a pre-addressed stamped envelope. A second packet was mailed to non-responders within six weeks of the first mailing. No remuneration was offered. A total of 583 (82%) eligible participants completed and returned the survey and form the basis of this report.

### Measures

Demographic and medical data were collected through the GELCC family registry database and medical records. Variables included age, gender, ethnicity, marital status, education level, body mass index (BMI), smoking status, number of first degree relatives with lung cancer, and time since lung cancer diagnosis of the affected family member(s).

The survey was developed to address the content areas described below by an expert panel, pilot-tested on 22 adults with cancer family history, then revised accordingly. The measures used were derived from studies that have proven to be reliable and valid tools assessing physical activity, nutrition, alcohol use, perceived cancer risk, degree of cancer worry/concern, and self-efficacy for behavioral change as well as for nutrition and exercise.

Health behaviors assessed were participants’ current levels of physical activity (PA) [34], nutrition [35], and alcohol consumption [36]. PA was measured via the Godin Leisure Time Exercise Questionnaire (GLTEQ) that has been shown to be a reliable and valid self-report measure [34]. This measure is comprised of three open-ended questions that assess the frequency of strenuous, moderate, or mild exercises for a period of at least 15 minutes over the last seven days. Scores higher than 24 units indicate moderate to strenuous PA – which meets public health recommendations for PA. Nutritional habits were assessed with a well-established, 23-item, Likert style measure (ranging from “never” [1] to “almost always” [5]) that asks over the past month how often participants had eaten certain foods such as butter, meat, or salad, with the option of not applicable [35]. The seven items that reflected negative eating habits (i.e., fast food, fried foods, red meat) were reverse scored. Mean scores were calculated for all 23 items. Higher scores indicated better nutritional habits while lower scores indicated poorer nutritional habits. Alcohol use was assessed using a two-part question modified from items originally on the Behavioral Risk Factor Surveillance System Questionnaire [36] that asked whether the participant had consumed at least 12 drinks of alcohol during their entire life. If they answered “yes,” then they were asked “on average, how many drinks do you usually have?” Six response categories were provided that ranged from “Less than one each month” (1) to “3 or more each day” (6).

Psychosocial characteristics were assessed via a combination of standardized scales with published psychometric properties and investigator-derived items from studies that have proven to be reliable and valid. Perceived cancer risk and degree of cancer worry/concern were single-item questions modified from items originally constructed by Lerman et al. [37]. On a 5-point Likert scale, respondents were asked, “How likely do you think it is that you will get cancer?” from “very likely” (1) to “very unlikely” (5) and on a 4-point Likert scale, “How concerned are you about getting cancer?” from “extremely concerned” (1) to “not at all concerned” (4). Degree of emotional closeness to affected family member(s) was measured by the question “How close is (or was) your relationship with this family member diagnosed with lung cancer?” which was adopted from the Texas Revised Inventory of Grief [38] shown to be reliable among family members. Structured on a 5-point Likert scale with responses from “closer than any other relationship I’ve had before or since” (1) to “Not very close at all” (5).

Self-efficacy for behavior change in general was measured via the General Self-Efficacy scale [39], a nine-item measure to assess one’s perceived ability to handle unforeseen situations, e.g., “I can always manage to solve difficult problems if I try hard enough.” The measure was scored on a 4-point Likert scale, with one being “strongly disagree” to four “strongly agree.” Scores ranged from 9–36; higher scores indicated more confidence while lower scores indicated lower confidence. Nutritional self-efficacy was assessed via the Nutrition Self-efficacy scale [40] measuring confidence to overcome barriers to making changes in eating habits. The measure was scored on a 4-point Likert scale, with one being “not at all confident” to four being “extremely confident.” Scores range from 5 to 20; higher scores indicated more confidence while lower scores indicated poorer confidence. Exercise self-efficacy was measured by a five-item measure designed by Marcus et al. [41] assessing confidence in one’s ability to engage in regular exercise in various situations, such as negative affect and making time for exercise (e.g., “When I am tired”). The measure was scored on a 10-point Likert scale, with one being “not at all confident” to 10 being “extremely confident.” Scores ranged from 5–50; higher scores indicated more confidence while lower scores indicated poorer confidence.

Respondents were asked investigator-generated questions assessing receptivity and preferences in cancer risk reduction lifestyle programs. Receptivity was measured by the question, “How willing would you be to take part in a lifestyle program (i.e., exercise, nutrition, smoking cessation) to help reduce your risk of getting cancer if we were to create one?” Response categories included “not at all,” “somewhat,” and “definitely.” Those responding, “somewhat” or “definitely” were asked five additional questions to assess delivery preferences. The first question was, “Would you want a program that was just for you or one that includes you and your family or others?” Categories of response were “Just me,” “Me and my family,” and “Me and others (i.e., people outside family like friends or co-workers).” Secondly, respondents were asked, “What type of lifestyle program(s) would be of interest?” with instructions to “Mark ALL that apply.” Choices included: exercise, weight management, nutrition, tobacco cessation (to quit smoking), stress reduction, and other, specify. Next, participants were asked to select their top choice from the types of lifestyle program(s) listed above. Participants were asked, “How likely would you be to take part if the program was delivered by phone, by mail, in person, by internet?” Categories of response were on a 3-point Likert scale ranging from “Very likely” to “Not at all likely.” Finally, respondents were asked to select their top choice for program delivery.

### Statistical Analysis

Descriptive data are presented as frequencies (n), percentages, means, and standard deviations (SD). Predictors of willingness to participate (“Definitely” or “Somewhat” willing versus “Not” willing) in a health promoting lifestyle program among family members was assessed using a generalized estimating equation approach to account for multiple family members who might participate from a given kindred [42]. For each variable of interest a univariable model was fit and then a final multivariable model was fit including variables that were significant in the univariable analyses. In all cases, p-values <0.05 were considered statistically significant.

## Results

### Respondent characteristics

Table 1 presents the demographic, behavioral, and psychosocial characteristics of the 583 respondents. Overall, respondents were older adults (mean age 65.3 years), Caucasian (98%), mostly female (64%), and highly educated (78% with more than 12 years of education). Eighty-eight percent of respondents had one or more first degree family member(s) affected by lung cancer. On average, the time since diagnosis of the most recently affected family member was 7.7 years (range: 0.5–19.5 years; SD=2.4 years).

When asked to describe overall health status, 85% of respondents reported being in good to excellent health. The mean BMI of respondents was 27.6 kg/m^2^ (range 17.0–46.3; SD=5.28) with 40% meeting criteria for being overweight (BMI=25–29) and 27% for being obese (BMI ≥ 30). Approximately half (54%) of the respondents reported engaging in moderate to strenuous physical activity (Godin score above 24). Respondents endorsed eating moderately healthy diets. A total of 45% of the participants indicated consuming alcohol weekly and 22% were current smokers.

With regard to psychosocial characteristics, 70% of respondents reported the degree of closeness with the family member diagnosed with lung cancer as closer than most relationships they had with others; 59% indicated directly being involved as a caregiver for a loved one with cancer. Among respondents, 38% perceived some risk for developing lung cancer in their lifetime and 58% perceived some risk for developing cancer in general in their lifetime. Moderate levels of lung cancer worry/concern and cancer in general were reported by 35% and 40% of respondents. Additionally, general self-efficacy scores indicated fairly high levels (mean=28.3; SD=4.21) of confidence in their ability to handle unforeseen situations. Similar results were seen in nutrition self-efficacy (mean=14.8; SD=3.06), respondents reflected fairly high levels of confidence in their ability overcome barriers to making change in eating habits. Moderate levels to engage in regular exercise during challenging situations were reported for exercise self-efficacy (mean=26.6; SD=10.66).

### Receptivity and preferences to a program

Table 2 shows self-reported willingness of respondents to participate in cancer risk reduction lifestyle programs. The majority of respondents (85%) were “Somewhat” or “Definitely” willing to participate in a lifestyle cancer-reduction program. Among those receptive to the programs, over half (56%) preferred to engage in a program with other family members. Preferred programs included weight management (36%) and nutrition (30%). The preferred modes of delivery were Web/Internet (45%) and mail (29%).

### Correlates of receptivity to participate

Univariable correlates among respondents who were receptive to lifestyle programs were female (p=0.026), had more formal education (p=0.016), and reported higher levels of perceived cancer risk (p=0.046) and concern about cancer in general (p=0.014) (Table 3). In addition, they endorsed higher levels of physical activity (p=0.02), lower levels of alcohol consumption (p<0.001), and higher levels of exercise self-efficacy (p=0.017). In a multivariable model of “definitely/somewhat willing to participate” versus “not,” the following variables were included in the model as potential predictors: gender, education level, physical activity, alcohol consumption, likelihood of getting cancer, concern of getting cancer, and exercise self-efficacy. From this model, higher exercise self-efficacy (p=0.025) was significantly correlated with interest.

## Discussion

This study provided important insight into the feasibility of using lifestyle cancer risk-reduction programs with a vulnerable population. The majority (85%) of the participants endorsed interest in cancer risk-reduction programs. Similar to previous research [23,24,28,29,31,43,44], these results emphasized that unaffected family members had a desire to improve and/or make health behavior changes to reduce their cancer risk. Moreover, our study participants expressed the most interest in weight management and nutritional programs – which are related and shown in the literature to be linked to reducing cancer risk [8,13,14,45]. This result is similar to that of Mazanec et al. [22] who found that family members of cancer survivors were interested in increasing physical activity and improving their nutrition. The potential benefits of positive changes in these health behaviors would not only help reduce cancer risk, but also reduce the risk of other chronic diseases and improve overall quality of life.

Concurrent with prior research [23,24], 65% of our study participants preferred a risk reduction program in a group-based format with their family members and friends. Typically, research on behavior change in cancer families has been focused either on the at-risk family members or on the cancer survivors. A natural source of support can produce a positive effect on one’s self-esteem, which, in turn, can increase motivation and retention to changes in behavior [46]. Moore et al. [47] found that among individuals at risk of diabetes, a group-based lifestyle program had a positive impact on diabetes knowledge, self-efficacy, increased physical exercise, healthier eating, and improvement in overall health. At-risk family members and cancer survivors should be studied as an integrated family unit to better understand and conceptualize a family or group-based cancer prevention program. Future studies could use a multi-dimensional assessment tool (e.g., Cancer Risk Belief Scale [48]) to explore individuals’ ideas about the role of family in cancer risk or employ qualitative methods to achieve a more in-depth understanding.

Family members preferred the delivery format of a program via web/internet (45%) and mail (29%) over other options. This preference could be attributed to many of the respondents being geographically dispersed. These formats would increase the reach and generalizability of such programs; for example, allowing for ease of information sharing between family members and friends. Internet-based programs could provide health promotion tailored to both the individual along with their family and friends.

On multivariate analysis, the receptive family members expressed greater exercise self-efficacy than those not receptive. Individuals with high/strong self-efficacy tend to persist with a behavior. It is possible that a lifestyle program implies the need to start an exercise program; thus, those receptive may have had greater confidence that this could be carried out. A weight management program would be beneficial to this group, given that 67% of those interested currently meet the criteria for obesity (BMI above 25) and 56% are not meeting the national recommendations for physical activity (Godin scores <=24). It is important for programs incorporating physical activity to address barriers such as lack of confidence in recruitment and intervention efforts. Interestingly, no other variables were significantly independently associated with receptivity to lifestyle programs such as timing of the patient’s lung cancer diagnosis, cancer worry, age, or gender.

An interesting observation is that among receptive family members, only 21% of those who smoked were interested in a tobacco cessation program. Recent studies have shown similar results in which smokers with a family history of lung cancer would like to quit at some point in the future but were not motivated to change their smoking behavior at that time [29,43,49–51]. Park et al. [51] found that lung cancer screenings for smokers had a harm reduction effect – such that post-screening, smokers tried to cut down but no one quit smoking. Regional differences may contribute to a decreased interest in smoking cessation programs. Those who live in the Midwest and South, especially women, have been found to have less interest in smoking interventions [4]. Age may be a factor that influences interest in smoking cessation. Kammin et al. [21] found that older individuals held more negative beliefs about smoking cessation and lung cancer susceptibility. This may explain why smoking cessation was not a priority in our sample of participants, given the fact that our sample was older. Those who smoke and who have done so for a significant amount of time may also have a fatalist attitude toward quitting; this may negatively impact their interest in making that lifestyle change [52]. Similarly, there are often conflicting media messages that “it’s never too late to quit,” which may allow smokers to rationalize delaying quitting [52]. In addition, current health status may also influence interest in smoking cessation. Park et al. [51] found that current smokers had little confidence in their ability to stop smoking to decrease their cancer risk and had little worry about smoking despite having perceived increased risk of developing the disease. It has been found that those who are in perceived poor health have less interest in smoking cessation programs, suggesting they may be prioritizing other health behaviors [21]. Within our study, family members who smoke expressed wanting to focus on changing other health behaviors to reduce their cancer risk – such as weight management and nutrition. Future studies could explore programs promoting multi-health behavior changes that could lead to motivation to change smoking behavior.

Strengths and limitations exist within this study. Strengths included the use of validated measures, large sample, and an existing family registry which made our study cost-effective and feasible to conduct in a controlled manner. Future studies should use a multi-dimensional manner of assessing (e.g., Cancer Risk Belief Scale; [48]) or qualitatively exploring the participant’s ideas about family and cancer risk. It would also be useful to assess if interest in lifestyle interventions is influenced by genetic vulnerability information obtained via genetic testing, since genetic testing is more available and better markers for lung cancer susceptibility have been identified [6,52]. In addition, this study assessed only theoretical receptivity to risk reducing programs and did not address if participants would indeed participate in actual programs. There is research suggesting that, for behavior change to be successful, it requires more than interest in making change and interest in decreasing cancer risk [21]. Those involving lifestyle interventions would need substantial support and education coupled with interest to maintain changes [21].

In conclusion, this study indicated individuals in cancer registries would be receptive to family-based lifestyle programs. Most seemed to prefer a method of delivery that utilized the Internet or mail, rather than face-to-face programs. These findings demonstrated that cost-effective public health programs may be highly beneficial for those types of individuals. Further, in-depth exploration is needed to determine what aspects of the cancer experience should be incorporated into lifestyle programs for cancer families that take into account their unique needs and concerns.

## Figures and Tables

**Table 1:** Genetic epidemiology of lung cancer consortium family member respondent characteristics (n=583).

	# (%)
**Age, years**	
N	580
Mean (SD)	65.3 (10.44)
Range	34.0–93.0
**Gender**	
Female	374 (64.2%)
Male	209 (35.8%)
**Education level**	
Elementary school or junior high	9 (1.6%)
High school/GED	119 (20.7%)
Some college/trade school	176 (30.7%)
College degree	165 (28.8%)
Postgraduate degree	105 (18.3%)
Missing	9
**Number of first degree relatives with lung cancer**	
1	511 (87.7%)
2	60 (10.3%)
3 or more	12 (2.1%)
**Time from lung cancer diagnosis of proband to survey completion by relative (years)**	
N	580
Mean (SD)	7.7 (2.4)
Range	0.5–19.5
**Smoke cigarettes**	
Never	108 (18.7%)
Former Smoker	325 (56.1%)
Current smoker	128 (22.1%)
Ever	18 (3.1)
Missing	4
Alcohol consumption	
**During your entire life, have you had 12 drinks or more of any kind of alcoholic drink?**	
No	40 (7%)
Yes	530 (93%)
Missing	13
Average drinks of alcohol	
None	40 (7.1%)
Less than one each month	152 (27%)
1 to 3 each month	107 (19%)
1 to 2 each week	86 (15.3%)
3 to 6 each week	103 (18.3%
1 to 2 each day	58 (10.3%)
3 or more each day	17 (3%)
Missing	20
**Body Mass Index, kg/m^2^**	
N	556
Mean (SD)	27.6 (5.28)
Range	17.0–46.3
<25	182 (32.7%)
25–30	224 (40.3%)
>30	150 (27%)
**Godin Score for physical activity**	
Mean (SD)	32.3 (36.8)
Range	0.0–640.0
<=24	258 (46.3%)
>24	299 (53.7%)
Missing	26
**Nutrition score**	
N	570
Mean (SD)	3.3 (0.55)
Range	1.7–4.7
**General self-efficacy score**	
N	574
Mean (SD)	28.3 (4.21)
Range	9.0–36.0
**Nutrition self-efficacy score**	
N	573
Mean (SD)	14.8 (3.08)
Range	5.0–20.0
**Exercise self-efficacy score**	
N	570
Mean (SD)	26.6 (10.66)
Range	5.0–50.0
**Overall health**	
**Compared to other people your age, how would you describe your state of health?**	
Excellent	86 (15.1%)
Very good	214 (37.5%)
Good	194 (34%)
Fair	64 (11.2%)
Poor	12 (2.1%)
Missing	13
Lung cancer risk	
**How likely do you think it is that you will get lung cancer?**	
Very likely	19 (3.3)
Somewhat likely	199 (34.3)
Somewhat unlikely	194 (33.4)
Very unlikely	100 (17.2)
I have no feeling or opinion on my chances of getting cancer	69 (11.9)
Missing	2
Cancer risk	
**How likely do you think it is that you will get cancer?**	
Very likely	53 (10.8%)
Somewhat likely	232 (47.2%)
Somewhat unlikely	124 (25.2%)
Very unlikely	33 (6.7%)
I have no feeling or opinion on my chances of getting cancer	50 (10.2%)
Missing	91
**Lung cancer worry/concern**	
**How concerned are you about getting lung cancer?**	
Extremely concerned	53 (9.1)
Moderately concerned	152 (26.2)
Mildly Concerned	258 (44.5)
Not at all concerned	117 (20.2)
Missing	3
**Cancer worry/concern**	
**How concerned are you about getting cancer?**	
Extremely concerned	44 (9%)
Moderately concerned	152 (31.1%)
Mildly Concerned	240 (49.4%)
Not at all concerned	53 (10.8%)
Missing	94
**Emotional closeness**	
**How close is (or was) your relationship with the family member diagnosed with cancer?**	
Closer than any relationship I’ve had before or since	115 (20.5%)
Closer than most relationships I’ve had with other people	276 (49.3%)
About as close as most relationships with others	130 (23.2%)
Not as close as most relationships	28 (5%)
Not very close at all	11 (2%)
Missing	16
**Caregiver**	
**Have you ever been directly involved as a caregiver for a loved one with cancer?**	
Yes	336 (58.5%)
No	238 (41.5%)
Missing	9

**Table 2:** Cancer risk-reduction program receptivity among lung cancer family members (n=583).

	# (%)
**How willing would you be to take part in a lifestyle program (i.e., exercise, nutrition, smoking cessation) to help reduce your risk of getting cancer if we were to create one?**	
Not at all	89 (15.3%)
Somewhat	289 (49.6%)
Definitely	205 (35.2%)
Missing	0
**If you answered somewhat or definitely, would you want a program that was just for you or one that includes you and your family or others?**	
Just me	169 (34.6%)
Me and my family	274 (56.1%)
Me and others (i.e., friends or coworkers)	45 (9.2%)
Missing	6
**Which of the programs listed above would be your top choice?**	
Exercise	80 (17.2%)
Weight management	168 (36.2%)
Nutrition	139 (30%)
Tobacco cessation	22 (4.7%)
Stress reduction	55 (11.9%)
Missing	30
**Of the four ways to deliver the program, which one would be your top choice?**	
Telephone	26 (5.3%)
Web/Internet	220 (45%)
In person	99 (20.2%)
Mail	144 (29.4%)
Missing	5

**Table 3:** Comparison of demographic, psychosocial and health behavior characteristics by lung cancer family members receptiveness to participate in lifestyle programs.

	Not Interested (N=89)	Somewhat or Definitely Interested (N=494)	p value*
Age			0.89
N	88	492	
Mean (SD)	65.3 (10.82)	65.3 (10.38)	
Range	(39.0–93.0)	(34.0–85.0)	
Gender			0.026
Female	47 (12.6%)	327 (87.4%)	
Male	42 (20.1%)	167 (79.9%)	
Education level			0.016†
Elementary school or junior high	2 (22.3%)	7 (77.7%)	
High school/GED	27 (22.7%)	92 (77.3%)	
Some college/trade school	28 (15.9%)	148 (84.1%)	
College degree	16 (9.7%)	149 (90.3%)	
Postgraduate degree	13 (12.4%)	92 (87.6%)	
Missing	3	6	
Number of first degree relatives with lung cancer			0.15
1	82 (16.0%)	429 (84.0%)	
2	5 (8.3%)	55 (91.7%)	
3 or more	2 (16.7%)	10 (83.3%)	
Time from lung cancer diagnosis of proband to survey completion by relative (years)			0.81
N	87	493	
Mean (SD)	7.8 (2.6)	7.7 (2.4)	
Range	(0.5–12.5)	(0.5–19.5)	
Smoke cigarettes			0.43
Never	20 (18.5%)	88 (81.5%)	
Quit	44 (13.5%)	281 (86.5%)	
Current Smoker	23 (18.0%)	105 (82.0%)	
Ever	2 (11.1)	16 (88.9)	
Missing	0	4	
Alcohol consumption			
On average, how many drinks of alcohol do you usually have?			<0.001‡
None	10 (25%)	30 (75%)	
Less than one each month	25 (16.4%)	127 (83.6%)	
1 to 3 each month	5 (4.7%)	102 (95.3%)	
1 to 2 each week	11 (12.8%)	75 (87.2%)	
3 to 6 each week	16 (15.5%)	87 (84.5%)	
1 to 2 each day	14 (24.1%)	44 (75.9%)	
3 or more each day	2 (11.8%)	15 (88.2%)	
Missing	6	14	
Body Mass Index (kg/m^2)			0.27
N	82	474	
Mean (SD)	28.2 (5.62)	27.5 (5.22)	
Range	(18.4–44.4)	(17.0–46.3)	
<25	12 (14.8%)	155 (85.2%)	
25–30	31 (13.8%)	193 (86.2%)	
>30	24 (16.0%)	126 (84.0%)	
Godin Score for physical activity			0.022
N	82	474	
Mean (SD)	26.7 (23.97)	33.8 (38.51)	
Range	(0.0–119.0)	(0.0–640.0)	
Score <=24	50 (19.4%)	208 (80.6%)	
Score >24	32 (10.7%)	267 (89.3%)	
Nutrition score			0.86
N	85	485	
Mean (SD)	3.4 (0.53)	3.3 (0.56)	
Range	(2.2–4.7)	(1.7–4.6)	
General self efficacy score			0.66
N	86	488	
Mean (SD)	28.5 (4.62)	28.2 (4.14)	
Range	(11.0–36.0)	(9.0–36.0)	
Nutrition self efficacy score			0.50
N	86	487	
Mean (SD)	14.5 (3.20)	14.8 (3.06)	
Range	(5.0–20.0)	(5.0–20.0)	
Exercise self efficacy score			0.017
N	84	486	
Mean (SD)	23.7 (12.34)	27.1 (10.28)	
Range	(5.0–50.0)	(5.0–50.0)	
Overall health			
Compared to other people your age, how would you describe your state of health?			0.13§
Excellent	18 (20.9%)	68 (79.1%)	
Very good	33 (15.4%)	181 (84.6%)	
Good	21 (10.8%)	173 (89.2%)	
Fair	11 (17.2%)	53 (82.8%)	
Poor	2 (16.7%)	10 (83.3%)	
Missing	4	9	
Lung cancer risk			
How likely do you think it is that you will get lung cancer?			0.51
Very likely	2 (10.5%)	17 (89.5%)	
Somewhat likely	25 (12.6%)	174 (87.4%)	
Somewhat unlikely	28 (14.4%)	166 (85.6%)	
Very unlikely	18 (18.0%)	82 (82.0%)	
I have no feeling or opinion on my chances of getting lung cancer	14 (20.3%)	55 (79.7%)	
Missing	2	0	
Cancer risk			
How likely do you think it is that you will get cancer?			0.046
Very likely	4 (7.6%)	49 (92.5%)	
Somewhat likely	27 (11.6%)	205 (88.4%)	
Somewhat unlikely	17 (13.7%)	107 (86.3%)	
Very unlikely	8 (24.2%)	25 (75.8%)	
I have no feeling or opinion on my chances of getting lung cancer	14 (28.0%)	36 (72.0%)	
Missing	19	72	
Lung cancer worry/concern			
How concerned are you about getting lung cancer?			<0.001
Extremely concerned	3 (5.7%)	50 (94.3%)	
Moderately concerned	12 (7.9%)	140 (92.1%)	
Mildly concerned	43 (16.7%)	215 (83.3%)	
Not at all concerned	31 (26.5%)	86 (73.5%)	
Missing	0	3	
Cancer worry/cancer			
How concerned are you about getting cancer?			0.014
Extremely concerned	2 (4.6%)	42 (95.5%)	
Moderately concerned	17 (11.2%)	135 (88.8%)	
Mildly concerned	37 (15.4%)	203 (84.6%)	
Not at all concerned	14 (26.4%)	39 (73.6%)	
Missing	19	75	
Emotional closeness			
How close is (or was) your relationship with the family member diagnosed with cancer?			0.29//
Closer than any relationship I’ve had before or since	15 (13%)	100 (87%)	
Closer than most relationships I’ve had with other people	35 (12.7%)	241 (87.3%)	
About as close as most relationships with others	21 (16.2%)	109 (83.8%)	
Not as close as most relationships	6 (21.4%)	22 (78.6%)	
Not very close at all	3 (27.3%)	8 (72.7%)	
Missing	5	11	
Caregiver			
Have you ever been directly involved as a caregiver for a loved one with cancer?			0.073
Yes	43 (12.8%)	293 (87.2%)	
No	44 (18.5%)	194 (81.5%)	
Missing	2	7	

GED = Graduate Equivalency Degree; SD = Standard Deviation.

* Adjusted P-value from Generalized Estimating Equations model for related family members.

† Comparing subjects with some college education versus others.

‡ P-value obtained is from combining 1 to 2 drinks each day and 3 or more drinks each day.

§ P-value obtained from combining fair and poor.

// P-value obtained is from combining responses “Closer than any relationship I’ve had before” and “Closer than most relationships I’ve had with other people” versus “About as close as most relationships with others” versus “Not as close as most relationships” and “Not very close at all”.

## Abbreviations

BMI: Body Mass Index
GELCC: Genetic Epidemiology of Lung Cancer Consortium
GLTEQ: Godin Leisure Time Exercise Questionnaire
PA: Physical Activity
SD: Standard Deviation
